# Acupuncture Treatment Reduces Incidence of Parkinson's Disease in Patients With Depression: A Population-Based Retrospective Cohort Study in Taiwan

**DOI:** 10.3389/fnagi.2020.591640

**Published:** 2020-12-04

**Authors:** Cheng-Hao Huang, Mei-Chen Lin, Ching-Liang Hsieh

**Affiliations:** ^1^Graduate Institute of Integrated Medicine, College of Chinese Medicine, China Medical University, Taichung, Taiwan; ^2^Department of Chinese Medicine, China Medical University Hospital, Taichung, Taiwan; ^3^Management Office for Health Data, China Medical University Hospital, Taichung, Taiwan; ^4^Graduate Institute of Acupuncture Science, College of Chinese Medicine, China Medical University, Taichung, Taiwan; ^5^Chinese Medicine Research Center, China Medical University, Taichung, Taiwan

**Keywords:** acupuncture, depression, incidence, Parkinson's disease, National Health Insurance Research Database (NHIRD), cohort study

## Abstract

Depression is a risk factor for subsequent Parkinson's disease (PD). Some patients with depression undergo acupuncture treatment because of other diseases in Taiwan. Therefore, the present study used data from Taiwan's National Health Insurance Research Database (NHIRD) to investigate the incidence of PD in patients having depression with and without acupuncture treatment. We conducted a retrospective study of a matched cohort of 48,981 patients with newly diagnosed depression between 2000 and 2012 who were selected from the NHIRD. The 1:1 propensity score method was utilized to match an equal number of patients (*N* = 9,189) in the acupuncture and non-acupuncture cohorts. We employed Cox proportional hazard models to evaluate the risk of PD. The cumulative incidence of PD in both cohorts was estimated using the Kaplan–Meier method, and the difference was examined through a log-rank test. Patients with depression who received acupuncture treatment demonstrated a lower risk of PD [adjusted hazard ratio (aHR) = 0.39, 95% confidence interval = 0.31–0.49] than those who did not undergo acupuncture treatment, after adjusting for age, sex, insurance amount, geographic region, urbanization levels, comorbidities, and drugs. The cumulative incidence of PD was significantly lower in the acupuncture cohort than in the non-acupuncture cohort (log-rank test, *p* < 0.001). The database did not indicate the severity of depression and acupoints. The results suggest that acupuncture treatment significantly reduced the development of PD in patients with depression; however, a future study should be conducted to provide more objective evidence.

## Introduction

According to the World Health Organization's (WHO) estimation, depression had the third highest global socioeconomic burden in 2008, and the ranking is estimated to increase to the first in 2030 (Malhi and Mann, 2018). Depression affected approximately 350 million people of all ages worldwide in 2012 (Marcus et al., 2012). Additionally, WHO estimated a 12-month prevalence of 3.2% for depression in 60 countries in 2003 (Moussavi et al., 2007; Kessler and Bromet, 2013). According to an epidemiological study in Canada, in 2002, the prevalence of depression was 5.0% in women and 2.9% in men, and after 10 years, the prevalence had increased to 5.8 and 3.6%, respectively (Albert, 2015). Several reports have indicated that patients with depression undergoing antidepressant treatment demonstrate increased incidence of Parkinson's disease (PD) (Alonso et al., 2009; Gustafsson et al., 2015). Moreover, studies have highlighted that depression is associated with stroke, cardiovascular disease, hypertension, diabetes (Thomas et al., 2004), and PD (Gustafsson et al., 2015). PD is a common and chronic brain degenerative disease caused by dopaminergic neurodegeneration in the substantia nigra pars compacta (SNpc) in the midbrain, which results in dopamine depletion in the striatum, causing motor symptoms (Kalia and Lang, 2015). Depression is thought to be a causal risk factor for PD (Shen et al., 2013; Leentjens, 2015), and studies have examined the association between depression and PD (Schuurman et al., 2002; Leentjens et al., 2013; Shen et al., 2013; Leentjens, 2015; Wang et al., 2018). A study found that depression may be an early precursor symptom of PD (Gustafsson et al., 2015). A retrospective cohort study indicated a strong relationship between depression and PD, and the hazard ratio (HR) with corresponding 95% confidence interval (CI) was 3.13 (1.95–5.01) for patients with depression compared with patients without depression (Schuurman et al., 2002). Thus, how to reduce the incidence of PD among patients with depression undergoing antidepressant treatment is crucial.

Acupuncture is a traditional and unique medical treatment in Asia. It has been in use for more than 2,500 years and is very popular due to its ease of administration, wide application, and rapid effects. Acupuncture is also an effective and safe treatment for depression, and after antidepressants, it is the second most popular treatment option for depression (Zhang et al., 2010). One study revealed that acupuncture treatment reduces the risk of stroke in patients with depression (Chen et al., 2019).

Since Taiwan's mandatory National Health Insurance (NHI) program was officially implemented in 1995, it has covered more than 98% of the population of Taiwan (Lee et al., 2010). The National Health Insurance Research Database (NHIRD) is a nationwide high-coverage database containing the data of insurants of the single-payer health insurance program in Taiwan. This database comprises medical information, including inpatient and outpatient records and prescriptions of western drugs and Chinese herbal medicines administered to each patient. An increasing number of people opt for Chinese medicine treatment in Taiwan because the treatment is covered by the NHI program (Wu et al., 2018). Because acupuncture treatment has been confirmed to be an alternative treatment option for several diseases, including stroke (Lu et al., 2017), depression (Zhang et al., 2010; MacPherson, 2014), and PD (Rajendran et al., 2001; Tamtaji et al., 2019). Depression affects most of patients with PD. In a clinical trial, acupuncture treatment decreases the depression scores and the Unified Parkinson's Disease Rating Scale (UPDRS) sub-scores among patients with PD (Yeo et al., 2018). Therefore, we'd like to clarify whether acupuncture reduces the risk of PD in depressive cohort. In the present study, we hypothesize that acupuncture treatment in patients with depression reduces the incidence of PD. Therefore, in this large population cohort study, the association between acupuncture treatment and the risk of PD was evaluated in 18,378 patients with depression from 2000 to 2012 identified from the NHIRD.

## Materials and Methods

### Data Source

The present study was conducted using data from the Longitudinal Health Insurance Database (LHID 2000), comprising 1 million patients randomly selected from the NHIRD. The identification details of study subjects were encrypted before the database was released for research. All historical diagnoses in the database were coded according to the International Classification of Disease, Ninth Revision, Clinical Modification (ICD-9-CM). The study was approved by the Research Ethics Committee of China Medical University and Hospital in Taiwan (CMUH-104-REC2-115-CR4).

### Study Population

**Figure 1:**
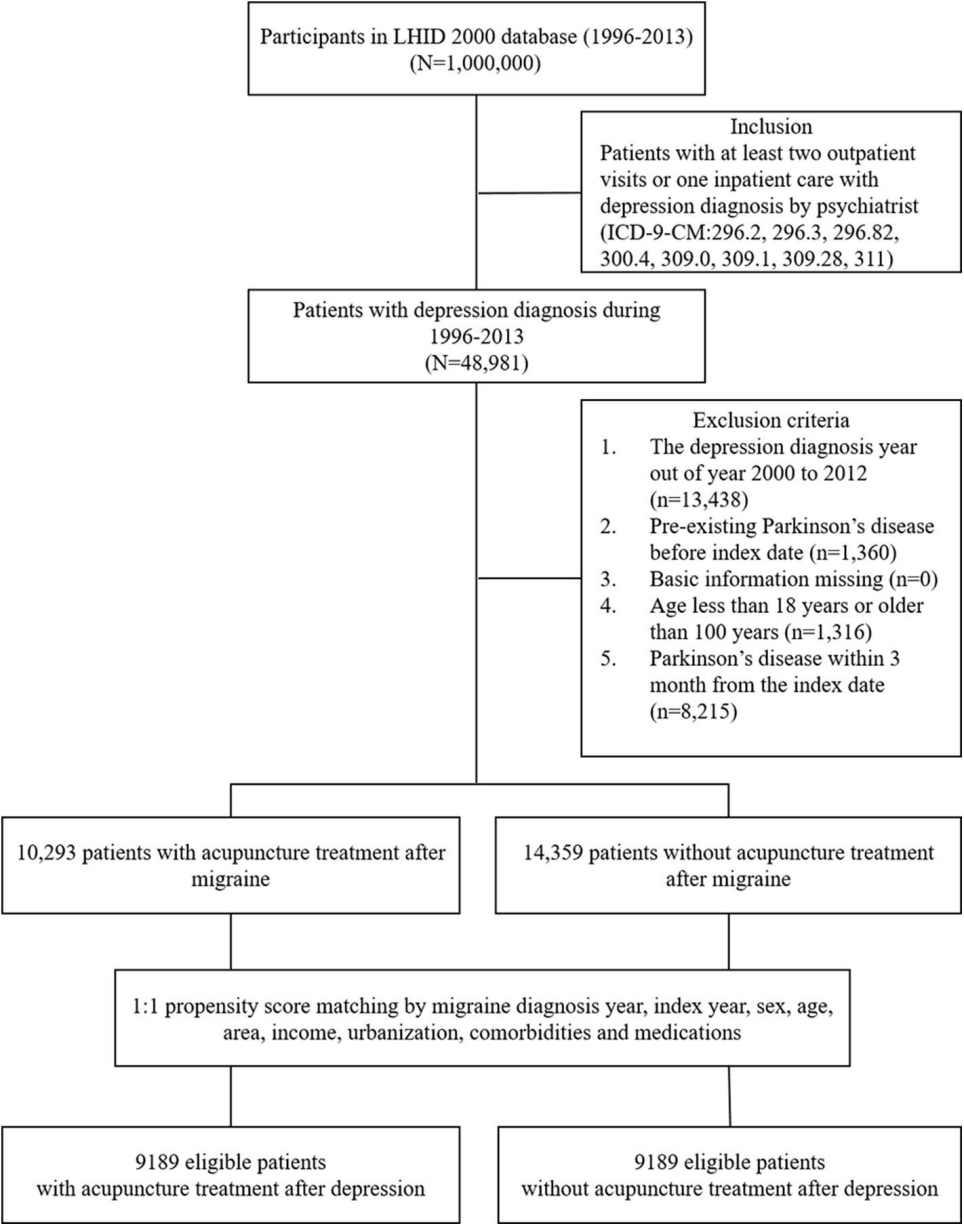
Study population flowchart. We identified 48,981 eligible newly diagnosed depression patients between 1996 and 2013. After using the 1:1 propensity score to match by sex, age, comorbidities, and drugs used, the groups of acupuncture users and acupuncture non-users each contained 9,189 patients. LHID 2000, Longitudinal Health Insurance Database 2000, NHI, National Health Insurance.

This study examined the risk of PD in patients having depression with or without acupuncture treatment, for which patients with depression were identified according to the following diagnostic codes: ICD-9-CM: 296.2, 296.3, 296.82, 300.4, 309.0, 309.1, 309.28, and 311. Depression cases were that were diagnosed by psychiatrists and involved at least two outpatient visits and one recorded hospitalization were included in the study. The case group included patients with depression had undergone acupuncture treatment after being diagnosed with depression. The acupuncture procedure codes were as follows: B41, B42, B45, B46, B80-B84, B90-B94, P27041, P31103, P32103, and P33031, and the following electroacupuncture (EA) codes were used: B43, B44, B86-B89, and P33032. The control group comprised patients having depression without any medical record of receiving traditional Chinese medicines and acupuncture treatment. The date of the first acupuncture treatment was set as the index date. Each case was 1:1 propensity score matched with a control by age, gender, depression diagnosis year, index year, comorbidities, and medications (Figure 1). The comorbidities comprised diabetes (ICD-9-CM: 250), hypertension (ICD-9-CM: 401–405), hyperlipidemia (ICD-9-CM: 272), congestive heart failure (ICD-9-CM: 398.91, 402.01, 402.11, 402.91, 404.01, 404.03, 404.11, 404.13, 404.91, 404.93, and 428), anxiety (ICD-9-CM: 300.0, 300.2, 300.3, 308.3, and 309.81), alcoholism (ICD-9-CM: 291, 303, 305.00, 305.01, 305.02, 305.03, 790.3, and V11.3), smoking (ICD-9-CM: 305.1), obesity (ICD-9-CM: 278), traumatic brain injury (TBI) (ICD-9-CM: 910, 850–854), and stroke (ICD-9-CM: 430–438), and medications included non-steroidal anti-inflammatory drugs (NSAIDs), oral steroids, statins, and antidepressants. The follow-up period was from the index date to PD diagnosis, withdrawal from the NHI program, or December 31, 2013.

### Statistical Analysis

Continuous and categorical variables were compared between the two cohorts using the *t*-test and chi-square test. To determine the risk of PD, Cox proportional hazard models were used to calculate HRs, adjusted hazard ratios (aHRs), and 95% CIs. Multivariate stratification analysis was performed to evaluate the risk of PD in different subgroups. The cumulative incidence of PD in the two cohorts was estimated using the Kaplan–Meier method, and the difference was tested through a log-rank test. All statistical analyses were performed using SAS statistical software, version 9.4 (SAS Institute Inc., Cary, NC, USA). The cumulative incidence curve was constructed using R software. A two-side *p* < 0.05 was considered significant.

## Results

Among the 18,378 eligible study subjects, 9,189 patients with depression had received acupuncture treatment (acupuncture group), and the remaining 9,189 patients with depression had never received acupuncture treatment (non-acupuncture group) (Table 1). Among all patients, 60% were female, and the mean age was 42.9 years. Gender, age, insurance amount, urbanization levels, comorbidities, and medications demonstrated no significant difference between the two groups after matching (*p* > 0.05); however, geographic region was statistically significant (*p* < 0.001). The majority of patients in the acupuncture group were treated with manual acupuncture (87.9%); 3.2% were treated with EA, and 9.0% received both. The mean number of acupuncture visits was 7.2 times during the study period.

**Table 1:** Characteristics of patients with depression accepted and non-accepted acupuncture.

**Variables**	**Accepted acupuncture**		*p*-value^*^
	**No (*n* = 9,189)** ***n* (%)/Mean (SD)**	**Yes (*n* = 9,189)** ***n* (%)/Mean (SD)**	
**Sex**			0.759
Women	5,852 (63.7)	5,832 (63.5)	
Men	3,337 (36.3)	3,357 (36.5)	
**Age at baseline**			
Mean(SD) (years)^†^	42.9 (16.7)	42.9 (15.6)	0.943
18–39	4,492 (48.9)	4,283 (46.6)	
40–65	3,606 (39.2)	3,978 (43.3)	
>65	1,091 (11.9)	928 (10.1)	
**Monthly income (NT$)**			0.502
0–15,840	4,876 (53.1)	4,808 (52.3)	
15,841–28,800	3,078 (33.5)	3,133 (34.1)	
28,801–45,800	885 (9.6)	920 (10)	
>45,800	350 (3.8)	328 (3.6)	
**Geographic region in Taiwan**			<0.001
Northern	4,444 (48.4)	4,346 (47.3)	
Central	1,931 (21)	2,225 (24.2)	
Southern	2,606 (28.4)	2,358 (25.7)	
Eastern	208 (2.3)	260 (2.8)	
**Urbanization**^**‡**^			0.641
1 (highest)	3,079 (33.5)	3,094 (33.7)	
2	2,918 (31.8)	2,872 (31.3)	
3	1,389 (15.1)	1,445 (15.7)	
4 (lowest)	1,803 (19.6)	1,778 (19.3)	
**Baseline comorbidity**			
Diabetes mellitus	1,533 (16.7)	1,531 (16.7)	0.968
Hypertension	2,703 (29.4)	2,696 (29.3)	0.910
Hyperlipidemia	2,559 (27.8)	2,561 (27.9)	0.974
Congestive heart failure	444 (4.8)	447 (4.9)	0.918
Anxiety	5,073 (55.2)	5,075 (55.2)	0.976
Alcoholism	415 (4.5)	415 (4.5)	1.000
Tobacco used	222 (2.4)	225 (2.4)	0.886
Obesity	177 (1.9)	183 (2)	0.749
Traumatic brain injury	1,100 (12)	1,109 (12.1)	0.838
Stroke	1,100 (12)	1,102 (12)	0.964
**Drug used**^¥^			
NSAIDs	74 (0.8)	72 (0.8)	0.868
Oral steroids	950 (10.3)	949 (10.3)	0.981
Statins	944 (10.3)	942 (10.3)	0.961
SSRIs	1,061 (11.5)	1,084 (11.8)	0.597
TCAs	1,044 (11.4)	1,031 (11.2)	0.762
Other antidepressant drugs	875 (9.5)	858 (9.3)	0.668
**Duration between depression date and index, days**	915 (868)	905 (908)	0.442
**Types of acupuncture**, ***n*** **(%)**			
Manual acupuncture of TCM type	–	8,073 (87.9)	
Electroacupuncture	–	291 (3.2)	
Combination of manual acupuncture and electroacupuncture	–	825 (9.0)	
**Acupuncture visits, times**	–	7.2 (15.1)	

* *Chi-square test*.

*t-test*.

‡ *The urbanization level was categorized into four levels according to the population density of the residential areas, with level 1 being the most urbanized and level 4 being the least urbanized*.

¥ *Drugs administered, comprising non-steroidal NSAIDs, oral steroids, statins, SSRIs (escitalopram, fluvoxamine, sertraline), tricyclic antidepressants (TCAs: amoxapine, desipramine, imipramine, doxepin, clomipramine, trimipramine), and other antidepressants [serotonin–norepinephrine reuptake inhibitors (SNRIs): venlafaxine, duloxetine, milnacipran; norepinephrine–dopamine reuptake inhibitor (NDRI): bupropion; serotonin antagonist and reuptake inhibitor (SARI): mesyrel; noradrenergic and specific serotonergic antidepressant (NaSSA): mirtazapine]*.

Table 2 presents the disease categories/diagnoses of patients who received acupuncture treatment. The most frequent diseases were injury and poisoning (ICD-9-CM: 800–999, 57.6%) and musculoskeletal system and connective tissue (ICD-9-CM: 710–739, 63.0%).

**Table 2:** Acupuncture cohort stratified by disease categories/diagnoses of patients with depression.

**Disease (ICD-9-CM)**	**Acupuncture users**	
	**(*****n*** **=** **9,189)**	
	***n***	**%**
Infectious and parasitic disease (001–139)	16	0.2
Neoplasms (140–239)	31	0.3
Malignant(140–208)	26	0.3
Benign (210–229)	7	0.1
Endocrine, nutritional and metabolic disease and immunity disorder (240–279)	55	0.6
Blood and blood-forming organs (280–289)	8	0.1
Mental disorder (290–319)	133	1.4
Nervous system (320–389)	518	5.6
Circulatory system (390–459)	193	2.1
Respiratory system (460–519)	296	3.2
Digestive system (520–579)	413	4.5
Genitourinary system (580–629)	167	1.8
Complications of pregnancy, childbirth, and the puerperium (630–676)	2	0.0
Skin and subcutaneous tissue (680–709)	69	0.8
Musculoskeletal system and connective tissue (710–739)	5,785	63.0
Congenital anomalies (740–759)	21	0.2
Certain conditions originating in the perinatal period (760–779)	0	0.0
Symptoms, signs and ill-defined conditions (780–799)	1,029	11.2
Injury and poisoning (800–999)	6,405	69.7

Table 3 presents the number of PD cases and HRs between patients having depression with and without acupuncture treatment. The risk factors for PD comprised increasing age (aHR = 3.19, 95% CI = 2.25–4.54; aHR = 9.79, 95% CI = 6.63–14.44), hypertension (aHR = 1.52, 95% CI = 1.13–2.03), TBI (aHR = 1.60, 95% CI = 1.22–2.10), and other antidepressant drugs (aHR = 1.83, 95% CI = 1.38–2.44). Undergoing acupuncture treatment (aHR = 0.39, 95% CI = 0.31–0.49), higher insurance amount (aHR = 0.45, 95% CI = 0.28–0.72; aHR = 0.42, 95% CI = 0.19–0.89), and use of oral steroids (aHR = 0.56, 95% CI = 0.37–0.85) and statins (aHR = 0.66, 95% CI = 0.48–0.90) were protective factors of PD.

**Table 3:** Cox proportional hazard models with hazard ratios and 95% confidence intervals of PD associated with acupuncture and covariates among patients with depression.

**Variables**	**No. of event (*n* = 355)**	Crude^*^		**Adjusted**^**†**^	
		**HR (95% CI)**	***p*-value**	**HR (95% CI)**	***p*-value**
**Accepted acupuncture**					
No	241	Ref.		Ref.	
Yes	114	0.41 (0.33–0.52)	<0.001	0.39 (0.31–0.49)	<0.001
**Sex**					
Women	216	Ref.		Ref.	
Men	139	1.08 (0.87–1.34)	0.484	1.17 (0.94–1.47)	0.159
**Age group**					
18–39	57	Ref.		Ref.	
40–65	141	3.01 (2.21–4.10)	<0.001	3.19 (2.25–4.54)	<0.001
>65	157	13.97 (10.32–18.92)	<0.001	9.79 (6.63–14.44)	<0.001
**Monthly income (NT$)**					
0–15,840	164	Ref.		Ref.	
15,841–28,800	165	1.53 (1.23–1.90)	<0.001	0.98 (0.78–1.23)	0.861
28,801–45,800	19	0.60 (0.37–0.97)	0.036	0.45 (0.28–0.72)	<0.001
>45,800	7	0.58 (0.27–1.24)	0.163	0.42 (0.19–0.89)	0.025
**Geographic region in Taiwan**					
Northern	167	Ref.		Ref.	
Central	68	0.82 (0.62–1.08)	0.158	0.80 (0.59–1.09)	0.158
Southern	111	1.14 (0.90–1.45)	0.287	0.98 (0.76–1.27)	0.883
Eastern	9	1.04 (0.53–2.03)	0.916	1.00 (0.49–2.01)	0.993
**Urbanization**					
1 (highest)	107	Ref.		Ref.	
2	127	1.23 (0.95–1.60)	0.108	1.21 (0.92–1.58)	0.173
3	43	0.85 (0.60–1.21)	0.370	0.86 (0.60–1.24)	0.415
4 (lowest)	78	1.19 (0.89–1.59)	0.242	0.97 (0.69–1.37)	0.877
**Baseline comorbidity (ref** **=** **non-site comorbidity)**					
Diabetes mellitus	119	2.76 (2.21–3.44)	<0.001	1.06 (0.82–1.36)	0.673
Hypertension	227	4.61 (3.71–5.73)	<0.001	1.52 (1.13–2.03)	0.005
Hyperlipidemia	154	2.22 (1.80–2.73)	<0.001	0.83 (0.65–1.06)	0.129
Congestive heart failure	54	4.04 (3.02–5.40)	<0.001	1.08 (0.79–1.48)	0.618
Anxiety	228	1.63 (1.31–2.03)	<0.001	1.11 (0.88–1.38)	0.382
Alcoholism	16	1.15 (0.70–1.90)	0.577	1.33 (0.79–2.25)	0.281
Tobacco used	4	0.57 (0.21–1.52)	0.257	0.66 (0.25–1.79)	0.420
Obesity	6	0.97 (0.43–2.18)	0.946	1.16 (0.52–2.62)	0.718
Traumatic brain injury	69	1.94 (1.49–2.53)	<0.001	1.60 (1.22–2.10)	<0.001
Stroke	118	4.12 (3.30–5.14)	<0.001	1.21 (0.93–1.56)	0.152
**Drug used**					
NSAIDs	4	1.03 (0.38–2.76)	0.952	1.71 (0.63–4.63)	0.288
Oral steroids	24	0.51 (0.33–0.77)	0.001	0.56 (0.37–0.85)	0.007
Statins	46	1.13 (0.83–1.54)	0.433	0.66 (0.48–0.90)	0.009
SSRIs	47	1.06 (0.78–1.43)	0.733	1.08 (0.79–1.48)	0.618
TCAs	49	1.10 (0.81–1.48)	0.546	0.82 (0.61–1.11)	0.207
Other antidepressant drugs	59	1.91 (1.44–2.52)	<0.001	1.83 (1.38–2.44)	<0.001

* *Crude HR represented relative HR*.

*Adjusted HR represented adjusted HR: mutually adjusted for accepted acupuncture, age, sex, income, geographic region, urbanization, diabetes mellitus, hypertension, hyperlipidemia, congestive heart failure, anxiety, alcoholism, tobacco consumption, obesity, TBI, stroke, oral steroids, NSAIDs, statins, SSRIs, TCAs, and other antidepressant drugs in Cox proportional hazard regression*.

Multivariate stratification analysis demonstrated decreasing risk of PD in different subgroups (Table 4), comprising female (aHR = 0.42, 95% CI = 0.32–0.56) and male (aHR = 0.38, 95% CI = 0.26–0.54) patients; those aged 18–39 years (aHR = 0.31, 95% CI = 0.17–0.57), 40–65 years (aHR = 0.43, 95% CI = 0.30–0.60), and more than 65 years (aHR = 0.36, 95% CI = 0.26–0.51); those with lower income (aHR = 0.34, 95% CI = 0.25–0.48; aHR = 0.42, 95% CI = 0.31–0.59; aHR = 0.24, 95% CI = 0.07–0.79); those living in northern (aHR = 0.37, 95% CI = 0.27–0.52), central (aHR = 0.39, 95% CI = 0.23–0.65), and southern Taiwan (aHR = 0.42, 95% CI = 0.28–0.64); those with all levels of urbanization (aHR = 0.37, 95% CI = 0.25–0.56; aHR = 0.34, 95% CI = 0.23–0.49; aHR = 0.48, 95% CI = 0.25–0.91; aHR = 0.45, 95% CI = 0.28–0.72); those with diabetes (aHR = 0.38, 95% CI = 0.26–0.56), hypertension (aHR = 0.45, 95% CI = 0.34–0.60), hyperlipidemia (aHR = 0.44, 95% CI = 0.31–0.61), congestive heart failure (aHR = 0.56, 95% CI = 0.32–0.97), anxiety (aHR = 0.41, 95% CI = 0.31–0.54), alcoholism (aHR = 0.17, 95% CI = 0.05-0.64), TBI (aHR = 0.37, 95% CI = 0.22–0.62), and stroke (aHR = 0.55, 95% CI = 0.38–0.80); those using statins (aHR = 0.36, 95% CI = 0.19–0.67), selective serotonin reuptake inhibitors (SSRIs) (aHR = 0.43, 95% CI = 0.23–0.82), and other antidepressant drugs (aHR = 0.46, 95% CI = 0.27–0.79).

**Table 4:** Incidence rates, hazard ratios, and confidence intervals of PD for patients with depression with and without acupuncture treatment stratified by sex, age, comorbidities, and drugs.

**Variables**	**Accepted acupuncture**						**Compared with non-acupuncture users**	
	**No**			**Yes**			**Crude HR**	**Adjusted HR**
	**(*****n*** **=** **9,189)**			**(*****n*** **=** **9,189)**				
	**Event**	**Person years**	**IR^†^**	**Event**	**Person years**	**IR^†^**	**(95% CI)**	**(95% CI)**
**Total**	241	36,182	6.66	114	42,008	2.71	0.41 (0.33–0.52)^***^	0.39 (0.31–0.49)^***^
**Sex**								
Women	145	22,575	6.42	71	26,108	2.72	0.43 (0.32–0.57)^***^	0.42 (0.32–0.56)^***^
Men	96	13,607	7.06	43	15,900	2.70	0.39 (0.27–0.56)^***^	0.38 (0.26–0.54)^***^
**Age group**								
18–39	43	19,136	2.25	14	19,752	0.71	0.32 (0.17–0.58)^***^	0.31 (0.17–0.57)^***^
40–65	89	13,557	6.56	52	18,156	2.86	0.45 (0.32–0.63)^***^	0.43 (0.30–0.60)^***^
>65	109	3,488	31.25	48	4,100	11.71	0.38 (0.27–0.54)^***^	0.36 (0.26–0.51)^***^
**Monthly income (NT$)**								
0–15,840	115	19,085	6.03	49	21,376	2.29	0.39 (0.28–0.54)^***^	0.34 (0.25–0.48)^***^
15,841–28,800	109	12,016	9.07	56	14,865	3.77	0.43 (0.31–0.59)^***^	0.42 (0.31–0.59)^***^
28,801–45,800	13	3,634	3.58	6	4,225	1.42	0.40 (0.15–1.05)	0.24 (0.07–0.79)^*^
>45,800	4	1,446	2.77	3	1,542	1.95	0.71 (0.16–3.15)	0.48 (0.07–3.54)
**Geographic region in Taiwan**								
Northern	115	17,125	6.72	52	19,262	2.70	0.41 (0.30–0.57)^***^	0.37 (0.27–0.52)^***^
Central	44	7,738	5.69	24	10,738	2.24	0.40 (0.24–0.66)^***^	0.39 (0.23–0.65)^***^
Southern	76	10,600	7.17	35	10,838	3.23	0.45 (0.30–0.68)^***^	0.42 (0.28–0.64)^***^
Eastern	6	717	8.36	3	1,170	2.56	0.36 (0.09–1.42)	0.28 (0.06–1.28)
**Urbanization**								
1 (highest)	72	11,774	6.12	35	13,716	2.55	0.43 (0.29–0.64)^***^	0.37 (0.25–0.56)^***^
2	88	11,531	7.63	39	13,118	2.97	0.39 (0.27–0.57)^***^	0.34 (0.23–0.49)^***^
3	29	5,522	5.25	14	6,656	2.10	0.40 (0.21–0.77)^**^	0.48 (0.25–0.91)^*^
4 (lowest)	52	7,355	7.07	26	8,519	3.05	0.44 (0.28–0.71)^***^	0.45 (0.28–0.72)^***^
**Baseline comorbidity**								
Diabetes mellitus	82	5,251	15.61	37	6,714	5.51	0.36 (0.24–0.53)^***^	0.38 (0.26–0.56)^***^
Hypertension	147	9,460	15.54	80	12,087	6.62	0.44 (0.33–0.58)^***^	0.45 (0.34–0.60)^***^
Hyperlipidemia	101	8,823	11.45	53	10,912	4.86	0.43 (0.31–0.60)^***^	0.44 (0.31–0.61)^***^
Congestive heart failure	32	1,411	22.69	22	1,848	11.90	0.53 (0.31–0.91)^*^	0.56 (0.32–0.97)^*^
Anxiety	152	18,581	8.18	76	21,817	3.48	0.43 (0.33–0.57)^***^	0.41 (0.31–0.54)^***^
Alcoholism	13	1,332	9.76	3	1,671	1.80	0.20 (0.06–0.69)^*^	0.17 (0.05–0.64)^**^
Tobacco used	3	682	4.40	1	766	1.31	0.30 (0.03–2.86)	0.33 (0.03–3.60)
Obesity	5	620	8.06	1	689	1.45	0.18 (0.02–1.51)	0.04 (0.00–1.03)
Traumatic brain injury	48	3,784	12.68	21	4,704	4.46	0.36 (0.22–0.61)^***^	0.37 (0.22–0.62)^***^
Stroke	69	3,535	19.52	49	4,755	10.31	0.54 (0.37–0.77)^***^	0.55 (0.38–0.80)^**^
**Drug used**								
NSAIDs	4	442	9.05	0	491	0.00	–	–
Oral steroids	16	4,874	3.28	8	5,374	1.49	0.46 (0.19–1.07)	0.44 (0.18–1.08)
Statins	31	4,261	7.28	15	5,073	2.96	0.40 (0.22–0.75)^**^	0.36 (0.19–0.67)^**^
SSRIs	31	4,692	6.61	16	5,381	2.97	0.45 (0.25–0.82)^**^	0.43 (0.23–0.82)^**^
TCAs	24	4,819	4.98	25	5,415	4.62	0.93 (0.53–1.62)	0.94 (0.53–1.67)
Other antidepressant drugs	37	3,402	10.88	22	3,987	5.52	0.51 (0.30–0.86)^*^	0.46 (0.27–0.79)^**^

*IR, incidence rates per 1,000 person-years; HR, hazard ratio; CI, confidence interval*.

*Adjusted HR represented adjusted hazard ratio: mutually adjusted for accepted acupuncture, age, sex, income, geographic region, urbanization, diabetes mellitus, hypertension, hyperlipidemia, congestive heart failure, anxiety, alcoholism, tobacco consumed, obesity, oral steroids, NSAIDs, statins, SSRIs, TCAs, and other antidepressant drugs in Cox proportional hazard regression*.

* *p < 0.05*;

** p < 0.01; and

*** *p < 0.001*.

**Figure 2:**
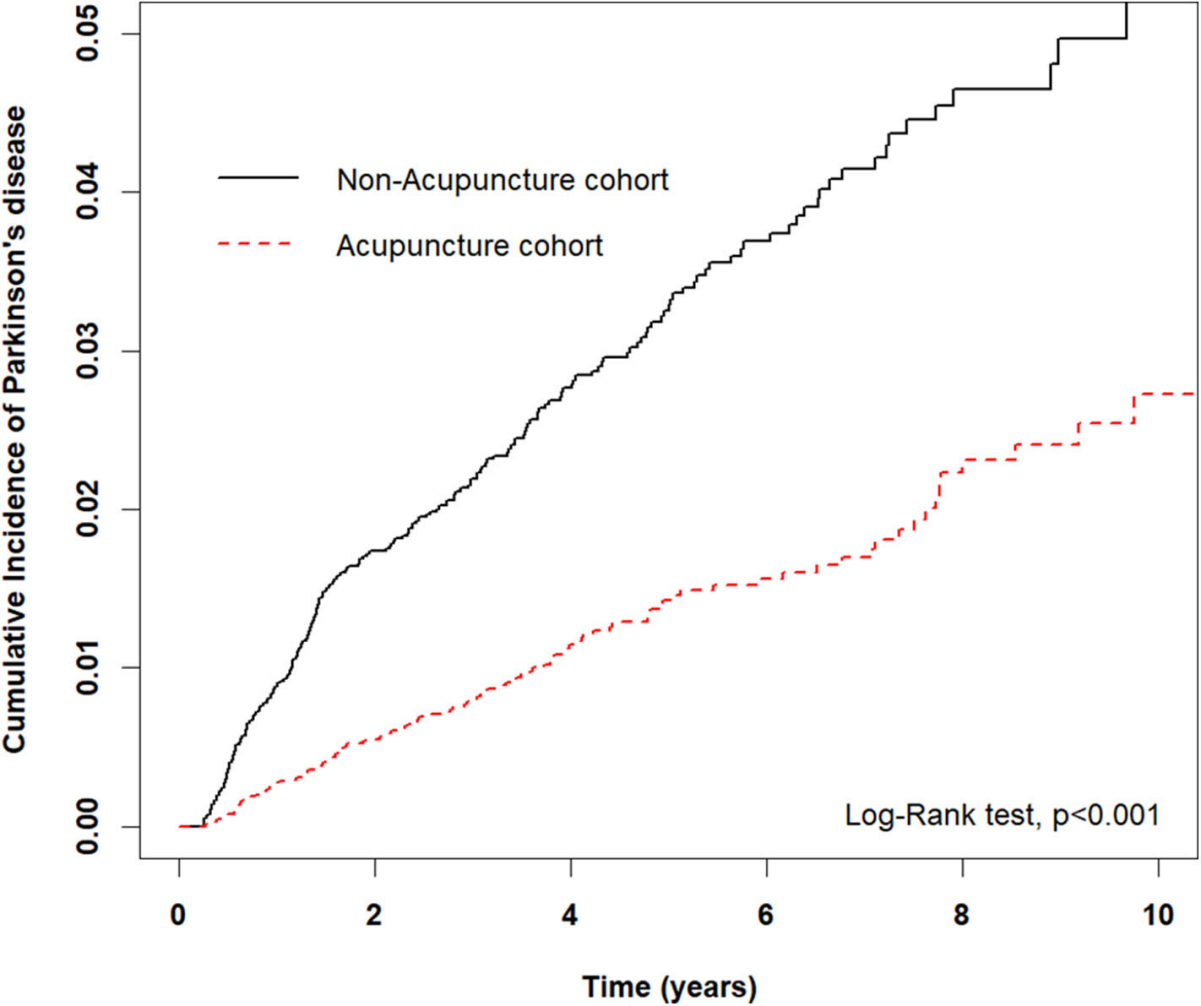
Cumulative incidence of PD between the acupuncture and non-acupuncture cohorts. The cumulative incidence of PD in the acupuncture cohort was significantly lower than that in the non-acupuncture cohort (log-rank test, *p* < 0.0001).

Figure 2 demonstrates significantly lower cumulative incidence of PD among patients having depression with acupuncture treatment than in those without acupuncture cohort (*p* < 0.001).

## Discussion

The results of the present study indicated that the incidence of PD was 61% lower among patients having depression with acupuncture treatment than among those without acupuncture treatment, suggesting acupuncture treatment decreases the risk of PD in patients with depression. After multiple adjustments for sex, age group, monthly income, geographic region, urbanization levels, baseline comorbidities, and drugs, acupuncture treatment was found to play a critical role in PD incidence reduction.

In our study, patients with depression who received acupuncture treatment were divided into two groups: those affected by injury and poisoning (57.6%) and those affected by musculoskeletal system and connective tissue disorders (63.0%). This results of correspondence with patients affected by injury and disease of the musculoskeletal system and connective tissue are the two major populations that undergo acupuncture treatment in Taiwan (Wu et al., 2018). TBI has been confirmed to increase the risk of depressive disorder (Chi et al., 2016), and head injury elevates the risk of depression by 59% (Orlovska et al., 2014).

Depression is the most common mental illness among PD patients. However, depression is also a risk factor for PD. Previous studies showed that acupuncture treatment would induce preventive effects in several disease populations associated with PD, including TBI (Li et al., 2017), stroke (Chen et al., 2019), hypertension (Terenteva et al., 2018), and diabetes mellitus (Shi et al., 2018). Therefore, we infer that acupuncture treatment also prevents from PD. This is the reason we conducted the study associated with the relationship between PD and depression with acupuncture treatment. In addition to affecting mental well-being and quality of life, depression is often associated with chronic musculoskeletal pain in patients. A study identified an elevated prevalence of depression in patients with chronic pain (Magni et al., 1990). Musculoskeletal system problems constitute another common disease found among patients with depression, which were treated with acupuncture in our study. Patients with depression often show symptoms such as muscle tension or soreness, sore bones and joints, headaches, and back pain. A study examined the association between depression and chronic pain in a general population in USA and discovered a high prevalence of depression symptoms among subjects with chronic pain (Magni et al., 1990). Generally, acupuncture is often adopted for treating most musculoskeletal problems of the four limbs, and EA is more effective for shoulder injuries (Cox et al., 2016). Furthermore, acupuncture is beneficial for treating chronic pain, and the relief effects of acupuncture may last up to 1 year (Vickers et al., 2018). A previous study identified the existence of abnormal connectivity in multiple brain regions, including cortical and subcortical areas, among patients with chronic pain (Apkarian et al., 2011), and acupuncture can regulate the activity of the pain matrix in various cortical and subcortical brain regions (Villarreal Santiago et al., 2016). Therefore, acupuncture can not only treat pain symptoms but also improve and activate specific areas of the brain.

PD occurs more commonly among elderly people, and the usual onset age is 50–79 years. PD is primarily a motor disease in which the nervous system gradually degenerates and results in decreased dopamine release in the brain. The present study also indicated that the risk of PD increases with advancing age. Patients aged 40–65 years demonstrated 3.19 times higher risk of PD, whereas the risk elevated to 9.79 times for those aged over 65 years. A study suggested that the incidence of PD in male patients increases till the age of 89 years (Driver et al., 2009). Accordingly, aging is recognized as a critical factor influencing PD incidence (Collier et al., 2017).

Of all baseline comorbidities associated with depression, hypertension significantly increases the risk of PD, except for TBI. Depression is closely related to changes in an individual's physical condition and may cause cardiovascular issues such as hypertension (Joynt et al., 2003). Patients with depression demonstrate a significantly increased risk of hypertension (Markovitz et al., 2001; Meyer et al., 2004). However, a meta-analysis suggested that hypertension is a risk factor for motor-stage PD (Hou et al., 2018), and it may influence PD patients' executive ability and memory (Jones et al., 2014). A previous study highlighted that up to 64.9% of patients with PD are affected by nocturnal hypertension (Tsukamoto et al., 2013). PD is considered to be a movement disorder caused by a massive loss of dopaminergic neurons in the SNpc. Chronic high blood pressure results in hypertensive vascular damage in several brain regions such as the basal ganglia and thalamus. This results in dysfunction and degeneration of dopaminergic neurons in the SN, in turn leading to dopamine transmission in the striatum (Qiu et al., 2011; Hou et al., 2018). However, acupuncture can be utilized as a combined treatment for alleviating hypertension and improving brain circulation and activation to maintain blood pressure and cognition (Sun et al., 2019).

The present study results also indicated that oral steroids and statin can decrease the risk of PD among patients with depression without acupuncture treatment. A study demonstrated that steroids can be used to treat central nervous system (CNS) diseases involving catecholamine by modulating brain activity and dopamine transmission (Sánchez et al., 2010). Steroids can not only protect neurons, glial cells, and blood vessels but also decrease the risks of affective disorders and PD (Garcia-Segura and Balthazart, 2009). In a national population-based study of patients with diabetes, statin users demonstrated a lower risk of PD than statin non-users did (Lin et al., 2016). Moreover, irrespective of whether simvastatin is used alone or in combination with metformin, the risk of PD is more reduced compared with when metformin is used alone (Brakedal et al., 2017). Additionally, a study of the 6-hydroxydopamine (6-OHDA) rat model, in which PD lesions were induced in animals, demonstrated the neuroprotective potential of statins against PD-like symptoms in rats (Kumar et al., 2012). Furthermore, a study showed that simvastatin halted dopaminergic neuronal loss induced by 6-OHDA in parkinsonian rat models and reported that simvastatin has neuroprotective potential against PD (Yan et al., 2011).

Increasing PD risk caused by depression can be attributed to several mechanisms such dopaminergic dysfunction, autonomic dysfunction, inflammation, and antidepressants (Lemke et al., 2004; Lee et al., 2013; Galts et al., 2019). Several studies have identified that dopamine, one of neurotransmitters in the brain, is associated with depression and PD (Lemke, 2008; Picillo et al., 2009). Compared with PD patients without depression, PD patients with depression have more frequent neuronal loss in the midbrain (Picillo et al., 2009). Moreover, lower dopamine levels were found in the brains of patients with TBI or hypertension (Yu et al., 1990; Lan et al., 2019). Acupuncture functions as a neuroprotector because it can increase dopamine levels in the brain and reduce brain atrophy by stimulating the acupoint of Baihui (GV20) in chronic cerebral hypoperfusion and ischemia-reperfusion injured rats (Chuang et al., 2007). Moreover, a study discovered that performing EA at Yanglingquan (GB34) and Taichong (LR3) acupoints could increase striatal dopamine levels in PD rodent models (Lin et al., 2017). Taken together, these results suggest that acupuncture may reduce the risk of PD in patients with depression, possibly by modulating dopamine function.

Except for hypertension, patients with depression are also critically affected by other cardiovascular diseases such as myocardial infarction and congestive heart failure. In other words, emotional problems may affect the autonomic nervous system (ANS) (Sgoifo et al., 2015). Therefore, ANS dysfunction resulting in vagal withdrawal facilitates the reduction of heart rate variability (HRV). Hence, compared with health subjects, reductions in HRV is reported in depression patients (Sgoifo et al., 2015). Autonomic failure in patients increases the risk of PD (Liepelt-Scarfone et al., 2015). Acupuncture not only alleviates autonomic responses by modulating the imbalance between sympathetic and parasympathetic activities but also stabilizes vagal activity in depression (Li et al., 2013; Noda et al., 2015). Several studies have reported that HRV increased significantly in patients with depression after acupuncture or EA (Shi et al., 2013, 2014). Acupuncture can reduce the risk of PD in patients with depression possibly partly from the modulation of acupuncture for ANS dysfunction.

Stress is associated with higher levels of proinflammatory cytokines, such as tumor necrosis factor-α (TNF-α) and interleukin (IL)-6, in patients with depression compared with healthy individuals (Galts et al., 2019). Several studies have shown increased levels of TNF-α, IL-6, and C-reactive protein (CRP) in patients with depression (Chen et al., 2016; Liu et al., 2019). Higher levels of proinflammatory cytokines in patients with depression are closely associated with a higher risk of PD. Both EA and acupuncture can significantly decrease TNF-α and IL-6 expression, which provides antidepressant effects and improves the hippocampal neuroinflammation in animal models of depression (Lu et al., 2016; Yue et al., 2018). In addition to TNF-α and IL-6, acupuncture significantly decreases the serum CRP level in another animal study (Qi et al., 2014). Therefore, acupuncture can reduce the development of PD in patients with depression, by possibly modulating the immune system.

Depression is associated with a decrease in monoamine neurotransmitters, including serotonin, norepinephrine, and dopamine; thus, the main pharmacological effect of antidepressants is that they enhance monoamine neurotransmission (Malhi and Mann, 2018). Several studies have reported that antidepressants increase the risk of PD. Both tricyclic antidepressants and SSRIs administered over 2–3 years may cause PD (Alonso et al., 2009; Zenesini et al., 2019). Several studies have reported that combining acupuncture and antidepressants is more effective than antidepressants alone over a 6-week period (Wang et al., 2014; Chan et al., 2015). A pilot study showed that acupuncture treatment using five acupoints (HT-7, LI-4, ST-36, SP-6, and LR-3) along with EA treatment using two acupoints (GV-20 and GV-24.5) is safe and effective for individuals with depression who are partially responsive or non-response to specific antidepressants (Yeung et al., 2011). To summarize, acupuncture reduces the risk of PD in patients with depression through multiple pathways, including the modulation of dopamine, ANS, immune system, and neurotransmitters.

The present study remains one question need to explanation; the mean number of acupuncture visits was 7.2 times only during the study period that is not enough to prevent the development of PD. We assume that acupuncture improves interest and enjoyment including in activities and increase the physical activity in patients with depression. Because regular physical exercise program can affect neuronal plasticity of the brain, repairing neuronal pathway before the occurrence of PD (Oliveira de Carvalho et al., 2018). The patients with depression are willing to receive another therapy aggressively; moreover, combining acupuncture and antidepressants may improve depressive disorder effectively (Chan et al., 2015) and then. In addition, no more than 15 visits of acupuncture treatment per month are allowed in Taiwan's NHI program. Some patients may receive additional acupuncture by self-pay which is not included in NHIRD database.

The present study had a few limitations. First, regarding data on depression, the NHIRD did not indicate the severity and duration of depression, and these factors possibly affected the incidence of PD. Second, the acupoints used in acupuncture treatments were not recorded in the NHIRD. Therefore, we were unable to clearly identify the acupoints that can treat depression and PD simultaneously. Third, the NHIRD does not contains laboratory data or brain images; therefore, no objective evidence could be found to explain the mechanism through which acupuncture reduces PD incidence in patients with depression, which warrants further study in the future. Fourth, because some PD cases may be late onset, the duration of the current study's follow-up period was insufficient. Double-blind, randomized clinical trials are required in the future.

## Conclusion

The present study results indicated that the incidence of PD was lower among patients with depression who received acupuncture treatment than among those patients with depression who did not, suggesting that acupuncture reduces the development of PD. This effect of acupuncture is mediated through multiple pathways, including the modulation of dopamine, ANS, immune system, and neurotransmitters. However, more objective evidence from clinical trials including laboratory data and brain images is required.

## Data Availability Statement

The datasets presented in this article are not readily available because due to the legal restrictions imposed by the government of Taiwan in relation to the Personal Information Protection Act, the data used for this study cannot be made publicly available. Request for data can be sent as a formal proposal to the NHIRD. Requests to access the datasets should be directed to http://nhird.nhri.org.tw.

## Ethics Statement

The studies involving human participants were reviewed and approved by the Research Ethics Committee of China Medical University and Hospital in Taiwan (CMUH-104-REC2-115-CR4). Written informed consent for participation was not required for this study in accordance with the national legislation and the institutional requirements.

## Author Contributions

C-HH conceptualized the study and drafted the manuscript. M-CL performed the statistical analyses. C-HH and C-LH contributed to the interpretation of acupuncture data and finalized the manuscript. M-CL and C-HH contributed to the interpretation of statistical data. All authors contributed to the article and approved the submitted version.

## Conflict of Interest

The authors declare that the research was conducted in the absence of any commercial or financial relationships that could be construed as a potential conflict of interest.

## Abbreviations

PD: Parkinson's disease
HR: hazard ratio
aHR: adjusted hazard ratio
CI: confidential interval
NHI: National Health Insurance
NHIRD: National Health Insurance Research Database
WHO: World Health Organization
SNpc: substantia nigra pars compacta
UPDRS: the Unified Parkinson's Disease Rating Scale
ICD-9-CM: International Classification of Disease, Ninth Revision, Clinical Modification
TBI: traumatic brain injury
NSAIDs: non-steroidal anti-inflammatory drugs
6-OHDA: 6-hydroxydopamine
HRV: heart rate variability
CRP: C-reactive protein
TNF-α: tumor necrosis factor-α
SSRIs: selective serotonin reuptake inhibitors
TCAs: tricyclic antidepressants
SNRIs: serotonin–norepinephrine reuptake inhibitors
NDRI: norepinephrine–dopamine reuptake inhibitor
SARI: serotonin antagonist and reuptake inhibitor
NaSSA: noradrenergic and specific serotonergic antidepressant.
